# Acquired Angioedema—A Challenge in Medical Practice: A Narrative Review

**DOI:** 10.3390/jcm15103800

**Published:** 2026-05-14

**Authors:** Katarzyna Poznańska-Kurowska, Małgorzata Skibińska, Dorota Lorenz, Waleed Aman Ur Rahman, Marcin Kurowski

**Affiliations:** 1 Department of Dermatology, Pediatric Dermatology and Oncological Dermatology, Dr. W. Bieganski Regional Hospital, Kniaziewicza 1/5, 91-347 Łódź, Poland; gosiaskibi@gmail.com (M.S.);; 2 Nofer Institute of Occupational Medicine, Clinic of Occupational Diseases and Environmental Health, Św. Teresy 8, 91-348 Łódź, Poland; 3 Department of Immunology and Allergy, Medical University of Łódź, Pomorska 251, 92-213 Łódź, Poland; waleed.amanurrahman@student.umed.lodz.pl; 4 GA2LEN/HAEi Angioedema Center of Reference and Excellence (ACARE), Department of Immunology and Allergy, Central Teaching Hospital, Medical University of Łódź, Pomorska 251, 92-213 Łódź, Poland

**Keywords:** acquired angioedema, hereditary angioedema, neoplasms, autoimmune diseases, complement C1 inhibitor protein, narrative review

## Abstract

Angioedema (AE) is a frequent symptom reported by dermatologists and allergists, as well as by general practitioners and physicians in other specialties. Hereditary angioedema (HAE) is an ultra-rare condition, whereas the majority of AE episodes in daily medical practice are secondary to an underlying condition or drug intake. This review discusses the most common causes of acquired angioedema, presents selected aspects of its pathogenesis in the context of available diagnostic tests, and provides an account of reports on possible management options. Acquired angioedema (AAE) poses an actual challenge both in terms of its diagnosis and management. Its variable etiology warrants a diagnostic approach aimed at the exclusion of underlying cancers, lympho- and myeloproliferative diseases, monoclonal gammopathies, as well as autoimmune and infectious conditions. Apart from its variable etiology, the management of AAE is further complicated by the lack of approved and standardized prophylaxis and treatment schemes. Therefore, an appropriate diagnostic approach is required for the efficient prevention of AAE symptoms and the detection of possible underlying pathologies.

## 1. Introduction

**Figure 1:**
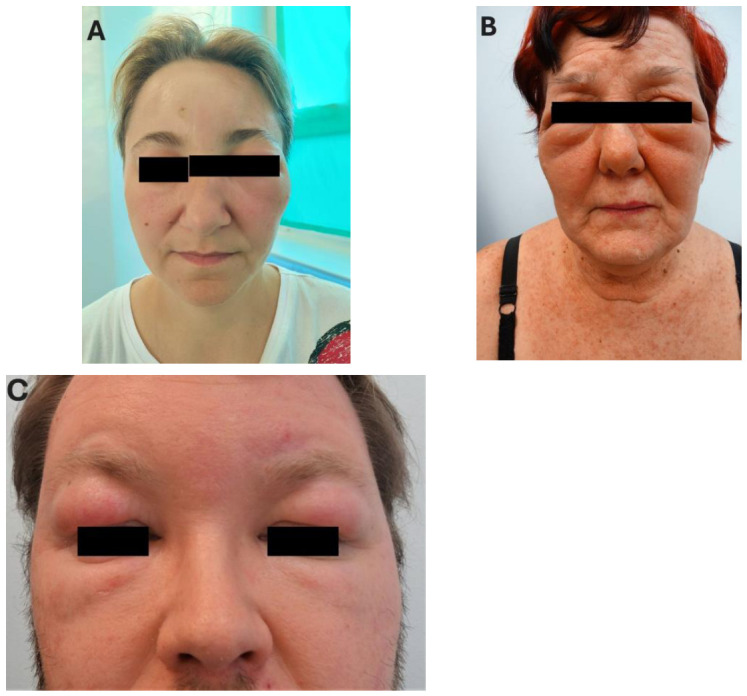
Angioedema symptoms located in the eyelids and periorbital areas: unilateral symptoms similar to those typical of HAE (**A**); symmetrical swelling of the eyelids warranting differential diagnostics with internal conditions (**B**,**C**). Photographs taken by the authors, patients consented to publication.

Signs and symptoms of angioedema are frequent reasons for patients seeking medical advice at general or specialist practices. Regarding the latter, dermatologists and allergists are the specialties most frequently confronted with patients presenting features of angioedema. Angioedema can be isolated or coexist with signs of urticaria [1,2]. Irrespective of the form of presentation, angioedema can always pose considerable difficulties and challenges in diagnosis and management. This is due to the heterogeneity in symptom localization, timing, and intensity, as well as the wide spectrum of conditions and pathomechanisms that may underlie the appearance of skin lesions [1,3,4,5]. In addition, symptoms suggestive of angioedema are a relatively common cause of seeking advice from medical professionals. The outer appearance of edematous swelling may not be characteristic and may share features of swelling attributable to numerous causes. In the context of a daily outpatient practice, swelling of the eyelids and periorbital areas is a common reason for referrals to dermatology or allergy consultations. In many cases, edematous lesions are symmetrical, suggestive of causes related to an acute allergic reaction as well as of being an external sign of internal disease (e.g., renal failure)—Figure 1A–C.

Additionally, abdominal symptoms—in particular if occurring in an isolated form—may be particularly misleading, and lead to unnecessary surgical interventions due to their clinical resemblance to acute appendicitis or peritonitis [6,7,8]. The genital area can also be affected, and swelling can occur as a result of sexual activity, but also, if caused by other stimuli, considerably impairs the quality of sexual life [9,10].

Hereditary angioedema (HAE) is a well-described entity that, however, belongs to the group of rare, genetically determined diseases and occurs with a frequency estimated at 1:10,000 to 1:50,000 subjects in the general population [2]. Various forms of acquired angioedema (AAE) are encountered far more frequently and are characterized by variable localization, intensity, and possible causes of symptoms [11,12].

Recently, a new classification of angioedema and its associated symptoms has been proposed within the framework of the DANCE (Definition, Acronyms, Nomenclature and Classification of Angioedema) initiative, formed by an international expert panel [13]. That classification takes into account both clinical features and underlying pathophysiological mechanisms of angioedema. Nevertheless, the rationale for such classification remains debated, and critical voices may be heard in the discussion, underscoring the premature nature of this approach at a time when the complex pathophysiology of angioedema is not yet fully elucidated, and several doubts and ambiguities remain [14].

Regarding both HAE and AAE, several forms have been identified based on their pathophysiology and, where applicable, genetic predisposition [15]. HAE may be due to decreased concentration and/or function of C1INH (types I and II, HAE-1 and HAE-2). In addition, several forms of HAE with identifiable genetic mutation yet normal C1INH level have been described [15,16,17]. Mutations in the *SERPING1* gene cause HAE-1 and -2, whereas genes, in which mutations have been described in HAE with normal C1INH include:

- Factor XII gene (*HAE-FXII*) [18];
- Angiopoetin-1 gene (*HAE-ANGPT1*) [19];
- Plasminogen gene (*HAE-PLG*) [20];
- Kininogen 1 gene (*HAE-KNG1*) [21];
- Myoferlin gene (*HAE-MYOF*) [22];
- Heparan sulfate 3-O-sulfotransferase 6 gene (*HAE-HS3ST6*) [23];
- *CPN1* (carboxypeptidase N1) [24];
- *DAB2IP* (disabled homolog 2-interacting protein) [25].

Acquired AE can also be due to decreased C1INH, not caused, however, by genetic mutations, and can result from the mechanism of action of angiotensin-converting enzyme inhibitors (ACEI), which are among the most commonly used antihypertensive drugs [26]. In the case of AAE, both histamine- and bradykinin-dependent (i.e., non-histamine-dependent) mechanisms may contribute to the development of clinical manifestations.

Histamine-mediated, or histaminergic, angioedema is currently being referred to and classified as “mast-cell mediated angioedema” due to histamine not being the sole mast cell-derived mediator released upon mastocyte stimulation by specific (e.g., allergic) or non-specific factors. In cases of specific IgE-dependent sensitization, the appearance of swelling results from the interaction of the allergen protein with immunoglobulin E (IgE) bound to the surface of a mast cell or basophil via the high-affinity receptor for the Fc fragment of an antibody (FcεRI). Binding, referred to as ‘cross-linking’, provides a stimulatory signal for the target cell, resulting in its degranulation and release of mediators. The secreted mediators are responsible for the early phase of the immediate IgE-mediated hypersensitivity reaction. Recent reports have added further complexity to the picture of AE pathogenesis, suggesting that mast cells may also be activated during HAE attacks, and that a cross-talk between mast cell-derived mediators and the complement system is possible [27,28]

In non-histamine-mediated angioedema, bradykinin, a potent vasodilator, contributes to increased vascular permeability and subsequent extravasation of fluid. Increased amounts of bradykinin (BK) may be a consequence of two mechanisms:

- Increased BK synthesis, or
- Decreased BK degradation.

Events that lead to increased synthesis of BK arise from reduced concentration or impaired function of C1-esterase inhibitor (C1INH) due to inborn or acquired conditions, as highlighted above. With C1INH deficient or absent, regulation and control of the contact system and the kallikrein–kinin pathways are insufficient. The contact system and kallikrein–kinin pathways are components of the plasma bradykinin-forming cascade. Therefore, the lack of C1INH-dependent regulation leads to increased bradykinin cleavage from high-molecular-weight kininogen (HMWK) [2,29].

This mechanism is involved in both hereditary and acquired angioedema. In addition, an excess of bradykinin can result from impaired degradation caused by medications that inhibit bradykinin-degrading enzymes, such as angiotensin-converting enzyme (ACE), carboxypeptidase N, neutral endopeptidase, dipeptidyl peptidase IV, or aminopeptidase P [29,30].

The current classification of angioedema, summarized above, remains a matter of debate due to expanding knowledge of potential new mechanisms of AE. Nevertheless, any form of angioedema is considered a diagnostic challenge, and reaching a solution may be time- and resource-consuming.

In this review, we aim to provide comprehensive insight into current clinical knowledge of acquired angioedema across various etiologies. AAE can be secondary to underlying conditions, in particular autoimmune disorders, monoclonal gammopathies, lymphomas, as well as solid tumors or induced by medications commonly prescribed in general practice (e.g., ACE inhibitors). In addition, it can be triggered by exercise and a wide spectrum of environmental stimuli [31].

Considering this, we decided to describe selected aspects of acquired angioedema, with a special focus on its most common and most significant underlying causes, likely to be encountered in the medical practice of various specialties.

## 2. Methods

Aiming at the identification of relevant sources concerning acquired angioedema for this narrative review, we have performed a literature search through PubMed and Scopus databases with the use of the following terms and strategy:

“acquired angioedema”
AND (pathogenesis OR mechanism)
AND (treatment)
AND (icatibant OR ecallantide OR “tranexamic acid” OR berotralstat OR lanadelumab OR garadacimab OR pd-C1INH OR rh-C1INH)

No restrictions on the publication date were applied. Subsequently, original articles, case reports, case series, and review articles were selected based on their titles and, when available, abstracts. Other potentially relevant papers were identified by manually checking the references of the included literature. The final selection of sources was agreed upon by all authors.

## 3. AAE of Known Etiology

### 3.1. Drug-Induced Angioedema

The spectrum of medical agents capable of inducing AE symptoms is wide; therefore, physicians of any specialty may be confronted with this form of AE in their practice. Drugs known to have an angioedema-causing activity include: angiotensin-converting enzyme inhibitors (ACEI), neprilysin inhibitors, dipeptidyl peptidase IV (DPP-IV) inhibitors, angiotensin receptor blockers (ARB), aliskiren (a direct renin inhibitor), recombinant tissue plasminogen activators (rt-PA), and statins [32]. Although structurally and mechanistically distinct, these medications share the ability to interfere with bradykinin function or metabolism at multiple levels.

Bradykinin—as mentioned earlier—is a product of the kinin–kallikrein system. It is cleaved from high-molecular-weight kininogen (HMWK) by the enzyme kallikrein. Acting through bradykinin 2 (B2) receptors, bradykinin affects vascular permeability and stimulates the release of substance P—a peptide that causes vasodilatation and fluid extravasation into tissues [33]. Bradykinin can be degraded by various kininases, with ACE and neprilysin being important in the development of angioedema symptoms [34].

Due to the long-established position of ACE inhibitors in the treatment of arterial hypertension, ACEI-induced AE is by far the most likely to be encountered in everyday practice and create diagnostic challenges, in particular if angioedema presents with non-specific symptoms [35,36,37,38]. Moreover, antihypertensives (including ACEI in a considerable percentage of cases) are predominantly prescribed in elderly patients, in whom already existing HAE tends to be less controlled and requires short-term prophylaxis more frequently [39]. Apart from its main action as an inducer of vasoconstriction and thus an elevation in blood pressure, ACE participates in the degradation of bradykinin. Hence, the induction of AE symptoms by ACE inhibitors results from their ability to decrease the degradation of bradykinin and other vasoactive agents, such as substance P [40,41]. The frequency of ACEI-induced angioedema is estimated at 0.2–0.3% of those who start treatment [33,42]. As indicated by data from a meta-analysis of 16 randomized controlled trials, treatment with ACEI increased the risk of AE symptoms by 2.8-fold compared with other active agents used as comparators [43]. Angioedema symptoms may appear at any time point during treatment with ACEI; however, in two-thirds of cases, the first symptoms appear within 3 months after the commencement of therapy [40]. To facilitate the diagnosis of ACEI-related angioedema and to provide a basis for lifetime ACEI avoidance recommendations, Bocquet et al. [44] proposed a diagnostic score that identifies patients with low or high probability of ACEI-related angioedema. For the identification of high-probability subjects, the reported sensitivity was 0.53, and the specificity was 0.97. Encouraging results of this raise hopes for better management and more justified avoidance recommendations in patients with ACEI-induced acquired angioedema. Furthermore, recent pilot research has aimed to identify genetic variants possibly related to ACEI-induced angioedema [45].

Data from various studies [46,47,48,49] identified several risk factors for the development of ACEI-induced AE, including African ethnic descent, female sex, oral contraception use, age ≥ 65 years, positive history of cardiac conditions, smoking history, drug-induced skin rash history, and seasonal allergies. In addition, DPP-IV inhibitors (e.g., gliptins, cyclosporin) and ±mammalian target of rapamycin (mTOR) inhibitors (e.g., sirolimus, everolimus) can increase the risk of AE symptoms if taken together with ACEI [26,33,50,51].

Angiotensin receptor blockers (ARBs), which are prescribed with similar frequency as antihypertensive treatment, do not block bradykinin degradation. However, AE associated with ARB treatment has been reported in meta-analyses and systematic reviews [42,52,53,54]. As reviewed by Suffritti and colleagues [32], the exact pathomechanism of ARB-induced angioedema has not yet been elucidated. It is supposed that ARBs may induce AE symptoms through indirect mechanisms. ARBs block the binding of angiotensin to its type 1 receptor (AT1R), thereby stimulating type 2 AT receptors (AT2R). AT2R stimulation, in turn, contributes to decreased ACE activity and increased bradykinin levels [55]. It has also been suggested that some ARBs (e.g., losartan) are partial agonists of the bradykinin B2 receptor, which may explain their ability to induce AE symptoms [56,57]. The overall rate of ARB-induced angioedema is considerably lower than that of ACEI-induced AE, estimated at 0.1%. Compared with ARBs, the risk of ACEI-induced AE is estimated to be 91% higher [33]. Most cases of ARB-induced angioedema have been related to either losartan or irbesartan [32,55,56], although AE symptoms seem to be a class effect rather than attributable to a single compound. Indeed, AE associated with candesartan [58,59], valsartan [60], and olmesartan [61] have also been reported.

Neprilysin inhibitors have been developed for administration in the treatment of chronic heart failure. Neprilysin prevents the disintegration of natriuretic peptides, mainly brain natriuretic peptide (BNP) and atrial natriuretic peptide (ANP). NEP inhibition increases the bioavailability of natriuretic peptides, which have beneficial effects on the cardiovascular system in heart failure—they increase natriuresis and diuresis and act as vasodilators. Neprilysin, or neutral endopeptidase (NEP), is localized in various tissues where, among other functions, it is an important player in the degradation of vasoactive mediators: bradykinin, substance P, and endothelin [26,34,62]. Pharmacological blockade of neprilysin turned out to be a risk factor for the development of AE symptoms. Not surprisingly, attempts to combine ACE and NEP inhibition in one medication (omapatrilat) significantly increased the risk of angioedema as compared to an ACE inhibitor (enalapril) alone [34]. Currently, a combination of a NEP inhibitor sacubitril with an ARB (valsartan) has been commercialized and is an available option for heart failure treatment. The main mechanism of action of sacubitril is to prevent the degradation of brain and atrial natriuretic peptides (BNP and ANP, respectively). NEP inhibition increases the bioavailability of natriuretic peptides. Subsequently, this increases natriuresis and causes vasodilation—both of which are highly beneficial in heart failure [34,62]. As reported recently by Eworuke et al. [63], the introduction of the sacubitril-valsartan combination in ACEI- and/or ARB-naïve subjects does not confer an increased risk of developing angioedema symptoms. This is, however, the case for previous ACEI or ARB users who have been switched to the sacubitril-valsartan combination.

Inhibitors of dipeptidyl peptidase IV (DPP-IV), also known as gliptins, are currently the mainstay of therapy for type 2 diabetes mellitus. DPP-IV is involved in the degradation of bradykinin and has an identified cleavage site (reviewed by Smolińska et al. [64]). Inhibition of DPP-IV may lead to the accumulation of bradykinin and another vasoactive peptide, substance P (SP). Increased risk of AE symptoms in susceptible patients is a sequel to insufficient degradation of bradykinin by other enzymes (aminopeptidase P, carboxypeptidase N, endopeptidase) Depending on the substance, the frequency of AE attributable to gliptin administration varies from ≥1:10,000 to less than 1:1000; however, in the case of sitagliptin, a relatively frequently prescribed DPP-IV inhibitor the actual risk of AE occurring as an adverse event cannot be established based on available reports [32]. Although the exact risk of AE during DDP-IV treatment has not been established, this class of drugs should receive closer attention as potential culprits. Increased vigilance is warranted, particularly in patients receiving concomitant treatment with other potential AE inducers, such as ACEIs [51]. This is further supported by the results of the recent pharmacovigilance analysis [61], which identified safety signals for AEs associated with both DPP-IV inhibitor monotherapy and combinations with drugs that interfere with the renin-angiotensin-aldosterone system (RAAS). The majority of those safety issues were identified with regard to various combinations of sitagliptin with RAAS modifiers, highlighting the apparent probability of drug-drug interactions in AAE pathogenesis.

Alteplase and tenecteplase are recombinant forms of tissue plasminogen activator (rtPA) and are widely used thrombolytics to treat ischemic events, predominantly stroke, but also acute myocardial infarction and pulmonary thromboembolism [61,62]. Although bleeding risk is the main concern during therapy with rtPA, angioedema incidents have also been reported, with an estimated frequency of 0.2 to 7.9%, and with predominant localization in oropharyngeal soft tissue, lips, and tongue [32]. The mechanism underlying angioedema after rtPA administration is associated with rtPAs’ role in hydrolyzing plasminogen to plasmin, which subsequently leads to bradykinin formation after cleavage of high-molecular-weight kininogen. In addition, activation of the factor XII and kinin–kallikrein system further contributes to AE formation. Similar to the AE cases associated with DPP-IV inhibitors, the risk of AE induced by rtPA increases if drugs interfering with RAAS are co-administered [65,66,67,68]. AE induced by rtPA occurs mainly in patients treated for ischemic stroke, as compared to other indications. Analysis of 151 cases presented by Maurier et al. [69] showed the percentage of stroke sufferers among patients with rtPA-associated AE to be as high as 98%. Additional factors associated with an increased risk of AE in rtPA-treated patients, according to analyses from various cohorts, include female sex, history of food or drug allergy, arterial hypertension, diabetes, and dyslipidemia [66,67,70].

Aliskiren, the first agent blocking the first step of the RAAS cascade via direct renin inhibition, was the first to be approved for antihypertensive therapy [71,72]. Although it is less frequently prescribed for the treatment of primary hypertension, as compared to ACEI and ARB, its role in generating angioedema symptoms has to be taken into consideration. Aliskiren participates in the modulation of the kinin–kallikrein system through an increase in kallikrein and bradykinin on the tissue level, the mechanism being considerably different from that of other RAAS inhibitors involved in the development of AE symptoms [73]. Increased bradykinin levels locally in the cardiac tissue have a cardioprotective action and reduce myocardial injury, the effect being mediated through the bradykinin B2 receptor [74,75,76]. From the point of view of its mode of action—tissular rather than plasma -located, aliskiren might be considered less likely to lead to angioedema symptoms [77]. Nevertheless, despite aliskiren’s mode of action seemingly distant from that attributed to ACEI and ARB, it may convey a considerable risk of the development of AE, irrespective of an individual’s presence or absence of previous episodes after other RAAS modifiers In a large database analysis, Toh et al. [78] assessed cumulative incidences, incidence rates, and hazard ratios for angioedema in adults treated with ACEI, ARB, or aliskiren, in comparison with those treated with β-blockers. Treatment with aliskiren was associated with comparable angioedema risk as treatment with ACE, while the risk was significantly lower in those treated with ARBs or β-blockers. In that study, however, the analysis of data on aliskiren-associated angioedema was based on 7 reported incidents among 4867 aliskiren-treated subjects. In contrast, Schlienger et al. [79], after analyzing 15,744 AE events among more than 3 million patients, of whom 3074 were on monotherapy with aliskiren, 1361 on a fixed-dose combination (FDC) therapy containing aliskiren, and 2238 on aliskiren free-standing combination (FSC) therapy, found the AE incidence rate in patients on aliskiren monotherapy or FDC to be comparable with that in subjects treated with β-blockers. These authors concluded that the increase in AE incidence observed among the group treated with aliskiren in FDC may rather be attributed to concomitant ACEI use. As shown in another database analysis [80], aliskiren-related AE events may differ in clinical features from those related to ACEIs or ARBs. Aliskiren-related AE was more frequently reported in females and tended to be more often peripheral and/or to involve the face and eyelids. Similar to ARB (and in contrast to ACEI), angioedema in aliskiren-treated subjects more frequently occurred during the first month of therapy. In view of the paucity of reported data, no conclusive information could be drawn with regard to the nature of aliskiren-associated AE episodes. However, in the majority of reports containing data on treatment, antihistamines or steroids were used. Hence, it may be speculated that a considerable percentage of aliskiren-induced AE could be histamine-mediated. Overall, the picture of aliskiren-associated angioedema may be blurred by infrequent prescribing, incompletely elucidated pathomechanisms, and concomitant use of other RAAS modifiers. Due to this fact, no conclusive recommendation regarding aliskiren as an alternative treatment for patients with ACEI-induced angioedema can be established.

Data regarding statins’ association with the occurrence of drug-induced angioedema symptoms are not numerous. Statins do not appear to interact directly with the kinin–kallikrein system; however, a hypothesis has been proposed, positing a possible involvement of statins in the increase in the number of bradykinin B2 receptors on the endothelial surface [22,59], thus favoring the development of AE symptoms upon bradykinin stimulation. Through their immunomodulatory effects, statins may hypothetically modify immune cell function and cytokine release, potentially resulting in local edematous lesions. However, these assumptions remain speculative, and the causal role of statins in the development of AE symptoms remains to be elucidated. Not many cases of angioedema that could be associated with statin intake have been described and published so far. The clinical picture of angioedema potentially associated with statin therapy varies considerably. Of importance, in some cases, it has co-existed with generalized urticaria, which adds to the possible complexity of mechanisms leading to AE symptoms. Cases of AE suspected of being statin-induced have been described with regard to lovastatin [81], pravastatin [81], atorvastatin [82,83,84], simvastatin [85,86], rosuvastatin [87], pitavastatin [88], and fluvastatin [89]. The question, however, remains open about the causative, and not only temporal, relationship between statin intake and angioedema. Doubts regarding this issue arise mainly from the considerable heterogeneity in the clinical picture of statin-related angioedema and its coexistence with urticarial hives, which may reflect involvement of mechanisms other than those directly related to bradykinin metabolism and the mechanism of action. In light of the center-stage role of statins in the management of dyslipidemia and their widespread prescription, the hitherto reported possible association of angioedema episodes with statin intake warrants planned observational studies in larger groups, more thorough and systematic case reporting, as well as basic mechanistic studies.

### 3.2. Acquired Angioedema with C1INHibitor Deficiency (AAE-C1INH)

AAE-C1INH shares multiple clinical features with hereditary angioedema (HAE) types I and II, except for the absence of genetic mutations in SERPING1 or other genes implicated in HAE pathogenesis [90]. Differential diagnosis of angioedema and classifying it as HAE or AAE creates considerable challenges and may be a source of pitfalls in managing patients with unexplained swelling.

AAE-C1INH is a rare, yet potentially serious and life-threatening condition characterized by recurrent episodes of angioedema, deficient C1INH inhibitor, and hyperactivation of the complement pathway [91]. It was first described in 1972 by Caldwell et al. [92] in 2 patients diagnosed with lymphosarcoma. Currently, the prevalence of AAE-C1INH is estimated at 1:100,000 to 1:500,000, approximately 10 times lower than that of HAE [91,93]. To present the matter more graphically, there is one patient with AAE-C1INH for ca. every 8 to 9 patients with HAE-C1INH, in accordance with data from German [91] and Italian [94] centers.

As mentioned above, AAE-C1INH can be a diagnostic challenge due to the similarity of its symptoms [95]. However, the age at first symptom onset may be an important clue when performing the differential diagnosis. In AAE-C1INH, the age of onset is higher than in HAE-C1INH. More than 90% of AAE-C1INH subjects experience their first symptoms after the age of 40, which is contrary to the early onset of HAE, where patients become symptomatic usually before reaching 20 years of age. The localization of symptoms during attacks in AAE-C1INH lacks features that clearly distinguish it from HAE. However, facial swellings tend to appear more often than extremities’ swellings [94].

Considering laboratory features, the main characteristic of AAE-C1INH is a decrease in C1q level, which is rarely seen in HAE. In the aforementioned reports [91,94], 88% and 81% of subjects had C1q levels below the lower reference range, with 59% and 50% of patients having C1q levels below 50% of normal, respectively. Therefore, C1q measurement is recommended as part of the diagnostic workup in patients with late-onset angioedema and decreased C1INH inhibitor levels [2,30,93,94,96].

**Figure 2:**
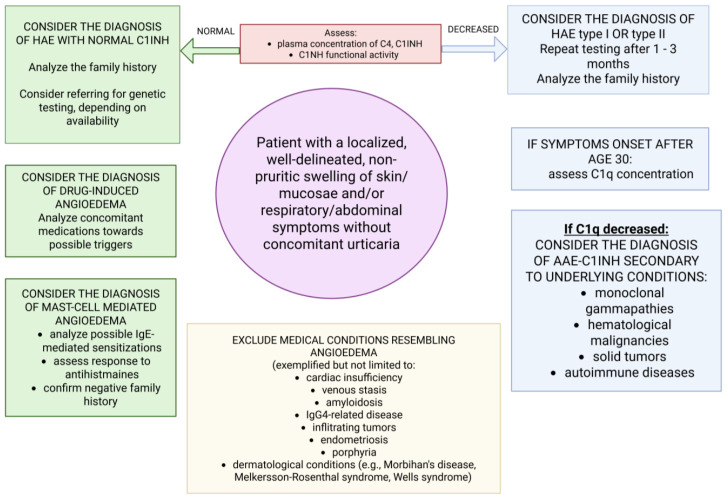
Key clinical considerations during evaluation and assessment of cases strongly suggestive of acquired angioedema. Based on information from [2,14,15]. Created in BioRender. Kurowski, M. (2026) https://BioRender.com/kq583c2 (accessed in 11 May 2026).

The appearance of new-onset angioedema in a person aged >40 should prompt a diagnostic workup to identify the underlying cause. A decrease in C1INH may be due to the presence of anti-C1INH autoantibodies resulting from a functional B-cell abnormality. C1 inhibitor deficiency in AAE-C1INH may also be secondary to increased consumption during conditions such as monoclonal gammopathy of undetermined significance (MGUS) or lymphoproliferative disorders. Still, autoimmune conditions (e.g., systemic lupus erythematosus, SLE) have also been ascertained in subjects with AAE due to C1 inhibitor deficiency [91,93,94,97,98,99,100,101]. It should also be noted that some cases of AAE-C1INH are not associated with any pathological condition, nor is the presence of anti-C1INH autoantibodies detected. In some cases, although no underlying condition or autoantibodies are confirmed, a diagnosis of MGUS or a lymphoproliferative disorder may be established during patient observation and follow-up [98,100]. Selected conditions associated most frequently with AAE-C1INH are listed in Table 1. In Figure 2, the main considerations for clinical and laboratory evaluation in recurring angioedema suggestive of AAE are presented.

## 4. Idiopathic AAE

### 4.1. Idiopathic Histaminergic AAE (AAE-IH)

Idiopathic histaminergic angioedema is a mast cell-related disorder whose symptoms result from the action of mast cell mediators. It can be clinically identified by a positive response to certain treatment modalities, both on an acute and chronic basis. Acute AAE-IH responds well to systemic antihistamines, glucocorticoids, and/or epinephrine. Its recurrence can be prevented by regular administration of oral second-generation antihistamines or omalizumab, the latter treatment being available in selected settings [102]. According to a commonly held belief, mast cell-mediated angioedema symptoms are usually accompanied by wheals typical of urticaria, but these two signs are not always observed concomitantly. Interestingly, Mansi et al. [103] retrospectively analyzed 1058 patients presenting with various forms of angioedema without wheals, of whom 379 were classified as having AAE-IH. Idiopathic histaminergic angioedema without wheals accounted for 35.8% of all cases of AE (i.e., hereditary and acquired) and 56% of cases of non-hereditary angioedema without wheals.

Although many clinical features overlap across multiple forms of AE, some distinctive characteristics have been described in idiopathic histaminergic angioedema without wheals. They include:

- Less frequent localization in the upper airways and gastrointestinal tract;
- Similarly to urticaria, AAE-IH develops rapidly, becoming pronounced within 30–60 min, usually resolving within 24 h after onset;
- In some cases, AAE-IH may be triggered by exposure to specific allergens (food, insect venoms, and others), physical stimuli, or infection. However, as the course becomes more chronic, specific inducers are less likely to be identified.

It should be underlined that many cases of AE resulting from mast cell mediator release are not IgE-dependent. In terms of suspected etiology, they resemble various forms of chronic spontaneous urticaria (CSU). Therefore, similar etiologies should be considered when diagnosing and differentiating isolated angioedema; however, most cases of histaminergic angioedema are accompanied by urticaria.

### 4.2. Idiopathic Non-Histaminergic AAE (AAE-InH)

The term “non-histaminergic angioedema” comprises AE subtypes that do not share the clinical features of AAE-IH, as presented above. Under this term, both hereditary and acquired forms of AE are included. Diagnosis of AAE-Inh can be established with a high degree of probability if several clinical features are present and possible underlying causes are ruled out (Table 2).

Description and comparative analysis of subjects with AAE-InH, HAE-C1INH, and HAE of unknown origin (HAE-UNK), provided by Andrási et al. [104], revealed that clinical characteristics of AAE-InH and HAE-UNK bear certain resemblances and that they differ in many ways from those encountered in HAE-C1INH subjects. The age of onset in AAE-InH is considerably higher (on average, 36 years vs. 29 years in HAE-UNK and 13 years in HAE-C1INH). Concerning symptom localization, facial and tongue edema occur more frequently. In contrast, edema in the upper airways, gastrointestinal tract, and distal parts of the extremities is found less frequently in the course of AAE-InH and HAE-UNK.

## 5. Treatment Options for AAE

AAE treatment may be equally challenging as its diagnosis. In contrast to HAE, in which many recognized drugs have been approved for prevention and treatment, no established treatment modality has been approved for AAE related to any known pathomechanism.

Such a situation results not only from widely variable pathogenesis and possibly dominant mechanisms (mast cell-mediated, bradykinin-mediated, idiopathic) but also from a lack of evidence-based uniform treatment strategies and recommendations. Such shortages and limitations are most evident in the case of AE presenting without wheals, for which urticaria-based treatment and prophylaxis strategies are not clearly indicated and are often ineffective.

If angioedema without wheals is seen in the non-specialist outpatient setting, it is most likely non-hereditary, given the rarity of occurrence of HAE, as presented in the introduction. Hence, a clear, easy-to-apply guideline would greatly facilitate preliminary classification and justify an initial treatment attempt in the primary care setting. Identification and, if applicable, introduction of an appropriate, tailored treatment for the primary disease underlying AE symptoms is the first and obvious step in the management of acquired angioedema secondary to autoimmune or inflammatory conditions, as well as to underlying cancers.

Despite the lack of a registered, approved scheme for AAE treatment or prophylaxis, multiple reports have been published describing the use of medications licensed for HAE with C1INH deficiency to treat AAE attacks of various etiologies. Of note, treatment of AAE attacks may be less responsive to C1-inh substitutional therapy due to frequent autoantibodies to C1-inh and accelerated complement protein consumption, particularly in AAE sequelae of lymphoproliferative conditions or autoimmune disease [105].

Currently available medications for HAE management are divided into long-term prophylaxis (LTP), short-term prophylaxis (STP), and on-demand treatment for acute HAE attacks. Their efficacy has been demonstrated in randomized clinical trials that meet Evidence-Based Medicine criteria, and they have been included in current guidelines and practice parameters [15].

Available on-demand HAE treatments include:

- Substitution of deficient C1-inhibitor with intravenous plasma-derived (pd-C1inh); or recombinant (rh-C1inh) preparation;
- Subcutaneous icatibant—an antagonist of B2 bradykinin receptor [106,107];
- Subcutaneous ecallantide—a kallikrein inhibitor [108,109];
- Newly FDA-approved oral kallikrein inhibitor—sebetralstat [110,111,112].

Short-term prophylaxis, recommended 1 to 6 h before exposure to a potential attack trigger (e.g., a surgical procedure or endoscopy), can be achieved by administering IV pd-C1inh or rh-C1-inh. However, in the latter case, the recommendations are not uniform worldwide. In case of restricted access to either C1inh preparation, the use of fresh frozen plasma or attenuated androgens (danazol, stanozolol) may be advised, with the understanding that its efficacy may be reduced.

Modern long-term prophylaxis modalities of HAE attacks include:

- Subcutaneous lanadelumab—a monoclonal antibody against plasma kallikrein [113,114,115,116,117,118,119,120];
- Berotralstat—an oral kallikrein inhibitor [120,121,122,123,124];
- Garadacimab—subcutaneously administered monoclonal antibody against activated factor XII [125,126,127,128];
- Subcutaneous pd-C1inh [129,130];
- Donidalorsen—a recently (August 2025) FDA-approved antisense oligonucleotide, specifically reducing prekallikrein expression [131,132,133].

### 5.1. Antihistamines in AAE Treatment

Antihistamines are very frequently the first-line drugs prescribed, somewhat ‘empirically’, by general practitioners in patients presenting with recurrent angioedema of undetermined etiology, with or without accompanying urticarial wheals. Their efficacy in recurrent angioedema has not been confirmed in randomized clinical trials. Nevertheless, a positive response to antihistamine treatment or effective prevention of recurrent angioedema with chronic antihistamine use may help identify mast cell-dependent, histaminergic angioedema among the cases of idiopathic recurrent swelling episodes [103,134]. Data from retrospective and observational studies differ in the frequency of anthistamine-responsive AE. In the group of 776 AE patients retrospectively analyzed by Zingale et al. [134], 294 were described as ‘idiopathic’, of which 86% (n = 254) responded well to antihistamine treatment. Among 1725 consecutive patients diagnosed with AE over almost a 20-year period, 449 had AE classified as ‘idiopathic’. Of them, 379 (84.4%) responded to long-term second-generation antihistamines and were therefore described as IH-AAE, while 70 histamine non-responders (15.6%) were diagnosed with InH-AAE [103]. A higher frequency of antihistamine non-responders among idiopathic AE cases has been described in a retrospective analysis of data from 120 patients with angioedema, selected based on medical records screening. Although a considerable percentage (64%) experienced benefits from long-term prophylaxis with an antihistamine at up to a quadrupled therapeutic dose, lack of improvement or even worsening of symptom intensity was observed in the remaining subjects [132]. Refractoriness to antihistamine therapy and prophylaxis should therefore be considered as a possible event during the management of recurrent idiopathic angioedema patients with an initial positive response.

### 5.2. Icatibant in the Treatment of AAE Attacks

In the context of HAE treatments employed for AAE management, reports on the use of icatibant—a bradykinin B2 receptor antagonist—are most numerous. Icatibant has been assessed in randomized controlled trials (RCTs) concerning its efficacy in the treatment of acute AE episodes induced by ACE inhibitors. Data from case series are available regarding icatibant efficacy in AAE induced by ACEI, as well as in AAE with decreased C1INH and various underlying pathologies.

#### 5.2.1. Icatibant in the Treatment of ACEI-Induced Angioedema

Baş et al. [135] compared the efficacy of 30 mg subcutaneous icatibant versus intravenous prednisolone (500 mg) plus clemastine (2 mg) in patients with ACEI-induced AE involving the upper aerodigestive tract, defined as face, lips, cheeks, tongue, soft palate or uvula, pharynx, and larynx. The primary endpoint was the median time to complete resolution of angioedema. Intensity and severity of AE symptoms were independently assessed by patients and blinded investigators on respective scales, and composite scores were calculated. Of the 27 patients analyzed, 13 received icatibant and 14 received prednisolone and clemastine. The median time to complete resolution of AE was 8.0 h (IQR, 3.0–16.0) in the icatibant group, compared with 27.1 h (IQR, 20.3–48.0) in the prednisolone/clemastine group (*p* = 0.002). None of the patients on prednisolone plus clemastine achieved complete resolution of symptoms within 4 h after treatment initiation, compared with 38% (n = 5) of patients who received icatibant (*p* = 0.02). The median time to the onset of symptom relief was shorter in the icatibant group than in the prednisolone/clemastine group—2.0 h (95% CI, 1.0 to 8.1) versus 11.7 h (95% CI, 8.0 to 18.0) (*p* = 0.03). In addition, changes in symptom intensity, as measured by patient- and investigator-performed assessment, following icatibant administration were considerably more pronounced than those resulting from therapy with prednisolone plus clemastine.

Sinert et al. [136] performed a multicenter RCT comparing the efficacy of 30 mg subcutaneous icatibant versus placebo in 121 subjects (61 receiving icatibant, 60 receiving placebo) with ACEI-induced angioedema lasting less than 12 h. The primary endpoint was defined as time to meeting discharge criteria, and secondary endpoints included: time to onset of symptom relief; occurrence of airway intervention, hospital admission; use of corticosteroids, antihistamines, or epinephrine for symptomatic relief after study drug administration; and number and proportion of subjects achieving the primary endpoint by 4, 6, and 8 h after study drug administration. No significant differences between icatibant and placebo were observed for the primary endpoint or any of the secondary endpoints established in this study.

In a randomized controlled trial involving a small number of patients (13 receiving icatibant, 18 receiving placebo), Straka et al. [137] also found icatibant ineffective in resolving angioedema attacks induced by ACEI treatment.

Additionally, Bova et al. [138] published a case series of ACEI-induced angioedema responding well to treatment with icatibant. They described 13 patients (among them 10 males) of age ranging from 52 to 86 years (median 74 years) who had been treated with icatibant for their angioedema not responsive to adrenaline, corticosteroids, and antihistamines. Alleviation of symptoms was reported at a median time of 30 min after icatibant administration (IQR 27.5–70 min), whereas complete resolution of symptoms was observed at 5 h (IQR 4–7 h) post-icatibant injection.

#### 5.2.2. Icatibant in the Treatment of AAE-C1INH

Longhurst et al. [139], in their Icatibant Outcome Survey study, reported on 16 patients with AAE and C1INH deficiency (AAE-C1INH), in whom 287 angioedema attacks had been treated primarily with self-administered subcutaneous icatibant injections. Data were collected by physicians over approximately 6 years (2009 through 2015) in 11 countries (Austria, Brazil, Denmark, France, Germany, Greece, Israel, Italy, Spain, Sweden, and the United Kingdom). In case of AAE-C1INH, as compared to HAE-C1INH, a visible, though not significant, tendency to localize more frequently on the face was observed (34.8% for AAE vs. 20.9% for HAE, *p* = 0.064). AAE-C1INH symptoms were less likely to run a severe or very severe course (40% vs. 61% in HAE-C1INH, *p* < 0.001) and more likely to respond favorably to icatibant administration. These beneficial icatibant effects on AAE-C1INH were observed earlier than in HAE, with time to symptom resolution after a single injection (2.3 h vs. 6 h in HAE, *p* = 0.031) and a shorter median attack duration (5 h in AAE vs. 9 h in HAE, *p* = 0.014).

Zanichelli et al. [140] reported successful icatibant administration in 8 patients with AAE-C1INH, among whom 4 had monoclonal gammopathy of undetermined significance (MGUS), and in 7, neutralizing anti-C1INH antibodies were detected. Overall, icatibant treatment has been reported for 48 attacks of moderate-to-severe intensity. Localization of attacks included pharyngeal/laryngeal site in 4 cases, face in 18 cases, and abdomen in 25 cases. One attack was limited to cutaneous symptoms. Additionally, two moderate attacks resolved spontaneously without treatment. A complete resolution of attacks could be attained within a median time of a little more than 9 h, ranging, however, from less than 2 h to 39 h. Notably, in 47 of 48 attacks, a single 30 mg subcutaneous dose of icatibant was sufficient to resolve symptoms. Icatibant effects became visible within half an hour, with a median time to onset of relief after icatibant administration ranging from 15 min to 21 h.

### 5.3. Other HAE Treatment Modalities Used in the Management of AAE

In addition to icatibant, several other treatments typically considered for HAE have been reported to be used in various settings to treat AAE. However, these reports are far fewer than those referring to icatibant.

Lanadelumab, an anti-kallikrein monoclonal antibody with an established role in long-term HAE prophylaxis, has been reported to be effective in subjects with AAE and low C1-inhibitor levels, in whom other treatment options (danazol, tranexamic acid) provided no improvement [141]. These observations from 3 cases showed that patients remained attack-free throughout the lanadelumab treatment period, ranging from 10 to 19 months. Suffritti et al. [142] reported a similar case of successful long-term prophylaxis with lanadelumab in a patient with transient IgM autoantibodies against C1-esterase inhibitor and a history of ineffective prophylaxis with tranexamic acid and danazol. Additionally, Gamboa et al. [143] described successful prophylactic administration of lanadelumab in a patient with recurrent life-threatening idiopathic non-histaminergic angioedema. Over a 2-year observation period, the number of attacks had been considerably reduced, and their localization had shifted from laryngeal to peripheral sites, posing no direct threat to the patient’s life.

Data on the efficacy of ecallantide, another kallikrein inhibitor, in AAE are even fewer. In a single report, Patel et al. [144] presented 3 patients with an acquired form of angioedema due to monoclonal gammopathy of undetermined significance (MGUS) in whom acute attacks could be successfully managed with IV ecallantide administration.

Finally, a real-world retrospective study [145] involving both patients with HAE (7 with HAE type I, i.e., with decreased C1-inh, and 2 with HAE with normal C1-inh) and AAE associated with C1-inh deficiency (n = 3) showed that prophylactic administration of berotralstat, an oral kallikrein inhibitor, led in all groups to reduction in number of angioedema attacks and to improvement in the disease control and in quality of life, as measured by angioedema control test (AECT) and angioedema quality of life (AE-QoL) questionnaires, respectively.

### 5.4. Omalizumab (OMA) in the Management of AAE Symptoms

As indicated above (see Section 3.1), an idiopathic histaminergic angioedema (AAE-IH) may share pathophysiological features with various forms of CSU. The involvement of IgE in mast cell degranulation and mediator release, as well as the guideline-established effectiveness of omalizumab in CSU, may prompt attempts to use OMA, an anti-IgE monoclonal antibody, in the long-term management of AAE. Several reports of cases of idiopathic angioedema with a suspected mast cell-dependent mechanism, which were successfully treated with OMA, have been published, as summarized by Zelin et al. [146]. These reports include patients unresponsive to up-dosed antihistamines and with AE symptoms not attributable to any biochemical abnormality, genetic trait, or underlying comorbidity. Treatment duration varied considerably between the reports, ranging from 2 months to 3 years. Similarly, the dosing regimens applied were not uniform. The most common dose was the same as in the approved CSU treatment schedule, i.e., 300 mg every 4 weeks; however, the doses and application intervals ranged from 75–150 mg every 3–8 weeks to 375 mg every 2 weeks. One of the reports included in Zelin et al.’s short literature review [146] provides data from a small randomized study by Goswamy et al. [147], which was designed as an exploratory, single-center, proof-of-concept trial. In that study, 10 patients were randomized 1:1 to receive omalizumab 300 mg subcutaneously or placebo every 4 weeks for 24 weeks. A follow-up was conducted over 12 weeks. The primary endpoint was the change in symptom intensity, as measured by the Angioedema Activity Score (AAS). In contrast, the secondary endpoints included changes in quality of life (measured with Angioedema Quality of Life Questionnaire (AE-QoL)), symptom intensity assessed on a visual analog scale (VAS), and the number of angioedema attacks reported per month. Significant improvement, as measured by Patient-Reported Outcome Measures (PROMs), was observed in AE symptom intensity and its impact on daily quality of life. This study, although carried out on a small sample of AE-affected subjects, provided promising results about OMA as a future therapeutic option for idiopathic angioedema. However, these results should be approached with caution due to the heterogeneity of symptomatology and pathophysiology of unexplained idiopathic angioedema. Special consideration should be given to the fact that IgE-mediated processes are not the sole feature of mast cell-mediated angioedema, whose pathomechanism extends far beyond the allergic reaction [148].

In light of currently available data on potential OMA effectiveness in isolated AE, the following issues remain unsolved and need to be further explored:

- Criteria for enrollment in OMA treatment—in particular:
  ▪ the degree of responsiveness or unresponsiveness of AE to quadrupled antihistamine dose, oral GCS, and epinephrine;
  ▪ coexistence of atopy features or symptomatic IgE-mediated allergy;
- OMA dosage and frequency of administration;
- Duration of treatment and, possibly, recommendations for dose tapering or extending the interval between injections;
- Potential usage of treatment effectiveness assessment tools (e.g., PROMs).

### 5.5. Tranexamic Acid in the Treatment of AAE

Tranexamic acid (TXA) has long been used for long-term prophylaxis of HAE, and its role in preventing HAE attacks remains important in settings with limited access to modern preventive modalities. Using TXA, an antifibrinolytic agent, for AAE prophylaxis may be feasible, given the activation of fibrinolysis and contact systems in AAE patients, particularly when autoantibodies against C1-inhibitor are present [149].

#### 5.5.1. TXA in the Management of AAE-C1INH

In a recent retrospective report, Kesh et al. [150] present a single-center experience treating 13 patients with AAE of various etiologies (among them 9 with AAE due to anti-C1INH antibodies) with TXA at a dose of 650 mg once or twice daily. During a 24-month follow-up, the number of attacks decreased from a mean of 18.36 ± 28.2 in the 12-month pre-observation period to 0.125 ± 0.4 over 24 months after TXA initiation.

In an Italian cohort of 101 patients with AAE-C1INH, Cancian et al. [151] found that long-term prophylaxis with tranexamic acid was effective in 31 of 36 patients receiving it chronically.

#### 5.5.2. TXA in the Management of AAE Induced by ACEI

TXA was also retrospectively assessed for its efficacy in the emergency treatment of ACEI-induced angioedema. A French study by Beauchêne et al. [152] included 33 patients treated at 2 tertiary French centers for ACEI-induced acute angioedema. In 27 of them, total improvement was achieved with TXA alone, while the remaining 6 patients showed partial improvement of angioedema and required additional treatment with icatibant or C1-inhibitor. None of the patients required intubation, and no fatalities were reported. A single-center retrospective study of 16 patients [153] set the proportion of patients who required intubation for suspected ACE-induced AE as the primary outcome. Except for 2 patients who required intubation before administration of TXA, no patient needed to be intubated after an intravenous administration of TXA at a dose of 1000 mg (or 100 mg in one case). In 85% of subjects, AE symptoms resolved completely or partially, without requiring ICU admission or further escalation of treatment. The average length of stay in the emergency unit was 3.5 h.

## 6. Future Perspectives and Actions in the Field of AAE

AAE, although presenting a vital clinical challenge, remains not fully elucidated and insufficiently addressed in hitherto published research. Substantial knowledge gaps can be identified concerning its epidemiology, risk factors, prophylaxis, and therapeutic management. These include, but are not limited to, the following issues:

- The prevalence of AAE across different etiologies should be the aim of studies involving larger populations from various ethnic backgrounds and defined by specific clinical characteristics.
- Symptoms of angioedema are frequently present in patients seeking consultations with dermatologists, allergists, general practitioners, and—in cases of ambiguous abdominal symptoms—gastroenterologists and surgeons. With this fact in mind, the underlying causes of AAE should be systematically and routinely considered in the differential diagnosis of medical emergencies across multiple specialties, and efforts should be made to establish uniform diagnostic pathways to accurately identify the conditions associated with AAE.
- Data on the efficacy of potential therapeutic options are based mainly on case series, except for icatibant, for which RCTs with limited numbers of subjects have been published, yielding contradictory findings. Therefore, a more thorough evaluation of potentially plausible treatment options, with a focus on those targeting the kinin/kallikrein/contact system, is warranted. Outcomes of such an evaluation would contribute to amendments to current practice parameters and the creation of new practice parameters and guidelines, supporting the increased accessibility of treatments currently available as off-label options.

## Figures and Tables

**Table 1:** Selected pathologies ascertained in patients with acquired angioedema due to C1-inhibitor deficiency (AAE-C1inh) and their reported frequencies (based on data published in: [91,93,94,98,100]).

Condition	Frequency in AAE-C1inh Cases
Monoclonal gammopathy of undetermined significance (MGUS)	40–47%
Non-Hodgkin lymphoma	20–28%
Presence of anti-C1-inh autoantibodies with no associated condition	9–18%
Systematic lupus erythematosus (SLE)	14–33%
No associated pathology	6–9%

**Table 2:** Selected clinical and laboratory features of idiopathic non-histaminergic angioedema (modified, after Andrási et al. [104]).

Negative family history of angioedema symptoms Normal C1-inhibitor concentration and activity Normal C4 concentration Absence of known genetic mutations responsible for HAE Absence of concomitant allergy and autoimmune disease (systemic and organ-specific) Absence of neoplastic disease (lymphoproliferative, myeloproliferative, solid tumors) Absence of chronic infection (viral, bacterial, parasitic, other) Lack of response to high-dose antihistamines and/or other conventional treatment (systemic glucocorticosteroids, epinephrine)

## Data Availability

No new data were created during preparation of this manuscript.

## Abbreviations

The following abbreviations are used in this manuscript:

AE	Angioedema
HAE	Hereditary angioedema
AAE	Acquired angioedema
C1INH	C1-esterase inhibitor
